# P01-06 Physical activity and non communicable diseases in France

**DOI:** 10.1093/eurpub/ckac095.006

**Published:** 2022-08-29

**Authors:** Roxane Berjaoui, Christele Gautier, Nathalie Joannard

**Affiliations:** 1 European and international affairs, French Ministry of Health and Prevention, Paris, France; 2 Sports direction, French Ministry of Sports and the Olympic and Paralympic Games, Paris, France

**Keywords:** Physical activity, non-communicable diseases

## Abstract

France, as a Member State of WHO Europe, plays a major role in the European strategy on physical activity, nutrition and health within a global framework for the ﬁght against non-communicable diseases.

France has adopted a National Sport Health Strategy for 2019-2024 intended to promote sports and physical activities (SPA) as a fully-fledged determinant of health and well-being lifelong. It is consistent with other strategies or plans implemented by other ministerial departments.

The four axes of the NSHS are: 1/Promoting health and well-being through physical activity and sports, 2/Developing the offer of and participation in adapted physical actvities for therapeutic purposes and prevention of loss of autonomy; 3/Better protecting sports people’s health and improving safety of activities whatever their intensity; 4/Improving and disseminating knowledge on the impacts on physical condition and health of engaging in sports and physical activities.

One of the key measure of the NSHS is indexing of “Maisons Sport Santé”, Health Sport-Houses (HSH) aimed at receiving and orientating all those wishing to engage in, develop or resume sport or physical activities for health or wellbeing reasons, whatever their age, state of health or frailty. Following 3 calls for projects, 436 HSH have been recognized since 2019 and cover almost whole French territory. HSH can be integrated within an association, a hospital, a sports establishment or as digital platforms. A particular attention is paid to people with highly sedentary lifestyles and those with limited autonomy. People with chronic or long-term diseases (cancer, diabetes, heart disease, asthma, Alzheimer etc.) to whom physical activities adapted to their limitations have been prescribed are also priority targets.

“Month of Sport and Physical Activities” is a large-scale communication campaign to promote the beneﬁts of sport and physical activities to a wide range of population. The aim is to spread recommendations on SPA and the ﬁght against sedentary behavior, to make SPA accessible to everyone and to encourage people and make them aware of the possibilities to practice in their daily life. Another goal is to give visibility to current actions in this area, to enable French people carry out an analysis of their physical condition and to spur them to (re) start a physical activity training.

